# Otx2 promotes granule cell precursor proliferation and Shh-dependent medulloblastoma maintenance in vivo

**DOI:** 10.1038/s41389-018-0070-6

**Published:** 2018-08-13

**Authors:** Salsabiel El Nagar, Almahdi Chakroun, Coralie Le Greneur, Dominique Figarella-Branger, Thomas Di Meglio, Thomas Lamonerie, Nathalie Billon

**Affiliations:** 1 Université Côte d’Azur CNRS, Inserm, iBV, Institut de Biologie Valrose, Nice Cedex 2, France; 2 Aix-Marseille Université, Inserm, CRO2 UMR_S 911, Marseille, France; 3 APHM, Hôpital de la Timone, Service de Radiothérapie, Marseille, France; 4 Centro de Biologia Molecular Severo Ochoa, Universidad Autonoma de Madrid, Madrid, Spain

## Abstract

The developmental gene *OTX2* is expressed by cerebellar granule cell precursors (GCPs), a cell population which undergoes massive expansion during the early postnatal period in response to sonic hedgehog (Shh). GCPs are thought to be at the origin of most medulloblastomas, a devastating paediatric cancer that arises in the developing cerebellum. *OTX2* is overexpressed in all types of medulloblastomas, except in Shh-dependent type 2 medulloblastomas, although it has GCPs as cell-of-origin. This has led to the current view that *OTX2* is not involved in tumorigenesis of this subgroup. How *OTX2* might contribute to normal or tumoral GCP development in vivo remains unresolved. Here, we have investigated, for the first time, the physiological function of this factor in regulating proliferation and tumorigenesis in the developing mouse cerebellum. We first characterized Otx2-expressing cells in the early postnatal cerebellum and showed that they represent a unique subpopulation of highly proliferative GCPs. We next performed in vivo loss-of-function analysis to dissect out the role of Otx2 in these cells and identified a novel, Shh-independent, function for this factor in controlling postnatal GCP proliferation and cerebellum morphogenesis. Finally, we addressed the function of Otx2 in the context of type 2 medulloblastomas by directing Shh-dependent tumour formation in Otx2+ cells of the developing cerebellum and assessing the effects of Otx2 ablation in this context. We unravel an unexpected, mandatory function for Otx2 in sustaining cell proliferation and long-term maintenance of these tumours in vivo, therefore bringing unpredicted insight into the mechanisms of type 2 medulloblastoma subsistence. Together, these data pinpoint, for the first time, a crucial Shh-independent role for Otx2 in the control of proliferation of normal and tumoral granule cell precursors in vivo and make it an attractive candidate for targeted therapy in Shh-dependent medulloblastomas.

## Introduction

Cerebellum development in mammals is characterized by postnatal transit amplification of granule cell precursors (GCPs), leading to tremendous numbers of granule neurons (80 billion in humans) in mature cerebellum^1^. In mice, GCP development proceeds along two phases: (1) between embryonic day 12.5 (E12.5) and 16.5, cells exiting the rhombic lip migrate at the surface of the cerebellum anlage to establish the external granule layer (EGL), and (2) after E16.5, GCPs proliferate intensely in the EGL up to postnatal day 15 (P15) before they exit the cell cycle and migrate inwards to take up their final position in the internal granular layer (IGL). GCPs proliferate in response to the mitogen Shh, secreted by the underlying Purkinje cells^2,3^. Shh binds to the trans-membrane receptor Patched (Ptch), relieving the Ptch repression of the G-coupled receptor Smoothened (Smo) and thereby allowing activation of the transcription activator Gli2 and inhibition of the transcriptional repressor Gli3^4^. Translocation of Gli2 into the nucleus induces the transcription of pro-proliferative genes, such as the cell cycle regulators Cyclin-D1, Cyclin-D2 and Cyclin-E^5^. Shh also stimulates the expression of the transcription factor N-Myc, which has recently been shown to exert a critical function in promoting GCP proliferation through the upregulation of cyclin-D2 expression^6–8^. Shh-induced extended proliferation of GCPs make them particularly vulnerable to malignant transformation and explains why they constitute the cell of origin for different groups of medulloblastomas^9^.

Medulloblastoma mainly arise in the developing cerebellum, with a peak of incidence at the age of 7. Treatments are very debilitating and despite some progress, the overall mortality rate remains high, reaching 50% for certain forms. Genetic and molecular analyses of these tumours have identified dysfunction of signalling pathways that promote mitogenic stimulation of cerebellar precursors or their growth arrest after transit amplification^10^. For instance, mutations in *PTCH1*, *SMO* or *SUFU* and somatic amplification of *GLI2* and *MYCN*, all deregulating Sonic hedgehog (SHH) signalling, define subgroup 2 medulloblastoma (or ‘SHH' subgroup). Murine models harbouring some of these alterations develop type 2 medulloblastoma, confirming that they constitute common drivers for tumour formation^10^.

Among recurrent genetic alterations found in medulloblastomas, one of the most frequent concerns the homeodomain OTX2 transcription factor. OTX2 is a developmental actor well known for its critical role in early central nervous system and sensory organ development^11^. *OTX2* is expressed by granule cell precursors throughout their massive expansion in the developing cerebellum^12^. Examination of large cohorts has revealed that *OTX2* is overexpressed in more than 75% of primary medulloblatomas and often correlates with classic histology and vermis localization^13^. The oncogenic role of OTX2 in the formation of cerebellar tumours has been supported by functional data, showing that silencing of *OTX2* expression in medulloblastoma cell lines reduces their proliferation potential in vitro and their tumorigenic properties^14–17^. Conversely, ectopic expression of OTX2 in immortalized epithelial cells or embryonic stem cell-derived neural precursors increases their proliferation and tumorigenicity^15,18,19^. Furthermore, ectopic expression of Otx2 in the mouse hindbrain induces hyperplasia in the cerebellum and brainstem, with cerebellar proliferative foci evocative of GCPs, suggesting that the oncogenic function of Otx2 in tumorigenesis could be attributed, at least in part, to its ability to stimulate GCP proliferation^20^. However, how Otx2 might influence GCP proliferation in vivo during normal cerebellum development and how it might contribute to medulloblastoma formation is currently unknown. Intriguingly, in contrast to group 3 and group 4 medulloblastomas, overexpression and focal gain of *OTX2* are usually not observed in type 2 tumours, where the Shh pathway is overactivated, even though this subtype is known to originate from GCPs^14,15,19,21,22^. This has led to the current view that Otx2 is not relevant in the tumorigenesis of Shh-medulloblastomas, although this has never been formally demonstrated.

The aim of this work was to investigate the physiological function of Otx2 in regulating GCP proliferation and tumorigenesis in the context of normal cerebellum development or type 2 medulloblastoma formation. Using unique mice models, we have identified a novel, Shh-independent, function for this factor in controlling postnatal GCP proliferation and cerebellum morphogenesis. Most importantly, our findings also unexpectedly highlight, for the first time, a mandatory role for Otx2 in sustaining long-term GCP tumoral proliferation and maintenance in type 2 medulloblastoma in vivo.

## Results

### Otx2-expressing granule cell precursors have an increased proliferative index

**Fig. 1:**
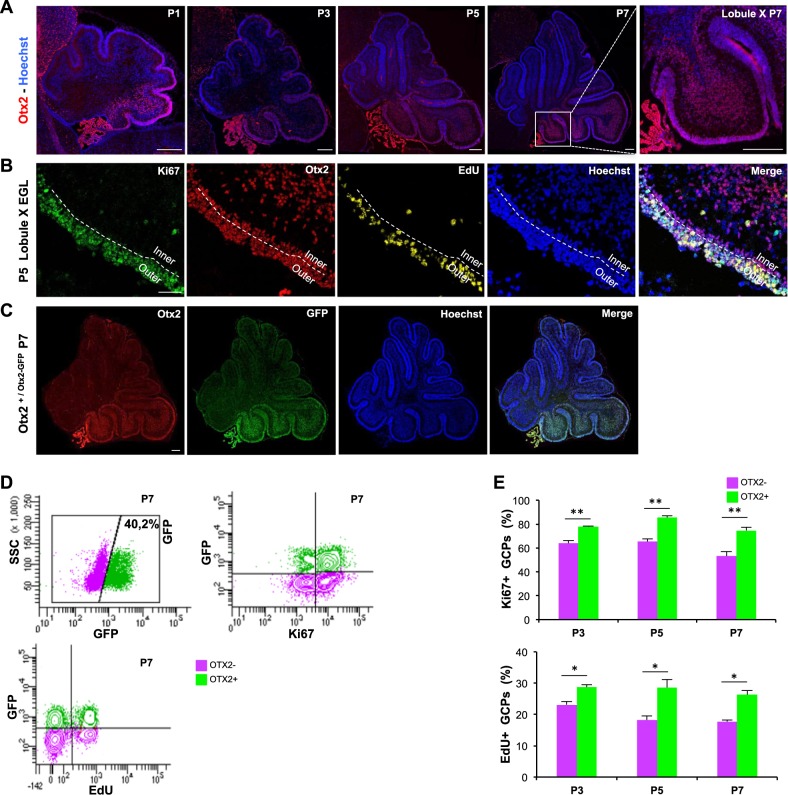
Identification and analysis of the proliferation potential of Otx2+ GCPs in the early postnatal cerebellum. **a** Expression of Otx2 in the posterior murine cerebellum at P1, P3, P5 and P7. Otx2 protein was detected by immunofluorescence staining (red) on cerebellar sagittal sections. Nuclei were counterstained with Hoechst (blue). Last picture on the right is an enlargement of lobule X at P7. **b** Detection of Otx2 and proliferating cells in lobule X at P5. Mice were injected with EdU 1 h before sacrifice. Ki67 (green) and Otx2 (red) were detected by immunostaining, while EdU (yellow) was revealed with a fluorescent azide. Nuclei were counterstained with Hoechst (blue). EGL can be subdivided into two parts (white dashed line): an outer proliferative zone and an inner non-proliferative zone. **c** Co-expression of Otx2 and GFP in the cerebellum of *Otx2*^*+/Otx2-GFP*^ transgenic mice at P7. Otx2 (red) and GFP (green) were detected by immunostaining, while nuclei were counterstained with Hoechst (blue). Merge of the two images shows a perfect overlap between Otx2 and GFP expression. **d** FACS analysis of GCPs purified from P7 *Otx2*^*+/Otx2-GFP*^ cerebella. GFP, Ki67 and EdU incorporation were detected by fluorescence staining on dissociated GCPs and analysed by FACS. Two populations of GCPs (Otx2+ and Otx2−) can be identified based on the expression of GFP. **e** Quantification of Ki67 expression and EdU incorporation in these two populations at P3, P5 and P7 indicate that Otx2+ GCPs have a higher proliferative potential than Otx2− GCPs. SSC side scatter, P postnatal day. Scale bar: 200 μm (**a**, **c**) and 50 μm (**b**). Data are represented as mean ± sem. *p < 0.05, **p < 0.005

We analysed Otx2 expression in postnatal mouse cerebellum between P1 and P7, since these stages cover the rise and peak of proliferation of granule cell precursors. Otx2 appeared restricted to the posterior cerebellum (Fig. 1a). It was strongly expressed in Ki67+/EdU+ proliferating GCPs of the outer EGL as well as in post-mitotic granule cells of the inner EGL and of the IGL, although to a lesser extent (Fig. 1b).

We next used a *Otx2*^*+*/*Otx2-GFP*^ knock-in mouse line to compare the proliferative potential of freshly purified Otx2+ and Otx2− GCPs (Fig. 1c). This line expresses a functional fluorescent Otx2– GFP fusion protein from the endogenous *Otx2* locus and recapitulates normal *Otx2* expression and activity^23^. Otx2+ and Otx2− progenitors could readily be separated by FACS analysis based on GFP expression, with Otx2+(GFP+) cells representing around 40% at P7 (Fig. 1d). Otx2+ GCPs showed a higher proliferative index, with a 20–40% increase of Ki67 expression and a 20–50% increase of EdU incorporation compared with their Otx2− counterparts (Fig. 1e), suggesting that Otx2 may act as positive regulator of GCP proliferation in the developing cerebellum.

### Otx2 cell autonomously controls GCP proliferation and development of the postnatal cerebellum

**Fig. 2:**
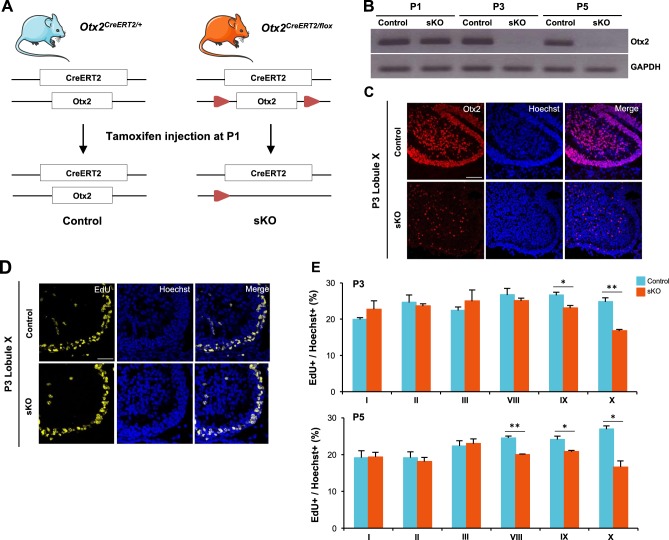
Effect of Otx2 ablation on GCP proliferation in the early postnatal cerebellum. **a** Schematic representation of the two *Otx2* alleles in *Otx2*^*CreERT2/+*^ (control) and *Otx2*^*CreERT2/flox*^ (sKO) mice, before and after tamoxifen injection. **b** Expression of *Otx2* RNA in control and sKO cerebella. *Otx2* RNA was detected by PCR 0, 2 and 4 days (P1, P3 and P5, respectively) after tamoxifen injection. **c** Expression of Otx2 protein in control and sKO cerebella. Otx2 was detected by immunostaining (red) on sagittal section of lobule X, 2 days (P3) after tamoxifen injection. Nuclei were counterstained with Hoechst (blue). **d** Detection of replicating cells in control and sKO cerebella at P3. Mice were injected with EdU 1 h before euthanasia and EdU incorporation was detected by fluorescence staining on sagittal sections of cerebellar lobule X (yellow). Nuclei were counterstained with Hoechst (blue). **e** Quantification of EdU-positive cells in the EGL of cerebellar lobules I, II, III (anterior), and VIII, IX and X (posterior) at P3 and P5, showing reduced numbers of proliferating GCPs in the posterior lobules of sKO cerebella. P postnatal day, sKO self-knockout. Scale bar: 100 μm. Data are represented as mean ± sem. *p < 0.05, **p < 0.01

To assess the function of Otx2 in the regulation of GCP proliferation, we examined the effects of *Otx2* ablation in the early postnatal cerebellum using a conditional self-knockout (sKO) *Otx2*^*CreERT2/flox*^ mouse line^12^. A 'non-floxed' *Otx2*^*CreERT2/+*^ line was used as control (Fig. 2a). We triggered *Otx2* sKO through a single pulse of tamoxifen at P1, which led to complete *Otx2* invalidation (Fig. 2b, c), and analysed its short-term effect on GCP proliferation (Fig. 2d). Quantification of EdU incorporation in posterior lobules (that expressed Otx2 before sKO) revealed a marked reduction in the number of proliferating GCPs in sKO mice at both P3 and P5 (Fig. 2d, e), with a 18%, 14% and 38% decrease in lobules VIII, IX and X at P5, respectively (Fig. 2e). In contrast, no significant change could be found in anterior lobules, which do not express *Otx2*.

**Fig. 3:**
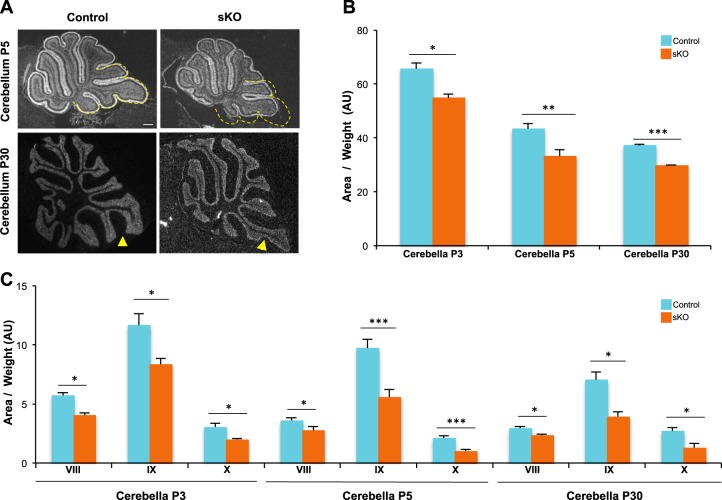
Effect of *Otx2* ablation on cerebellum expansion and foliation at perinatal and adult stages. **a** Morphogenesis defects in *Otx2* sKO cerebellum at perinatal and adult stages. Mid-sagittal sections of control and sKO cerebella were stained with Hoechst to visualize their structure and foliation at P5 and P30. Yellow dotted line indicates the size of the control posterior cerebellum. Yellow arrows show the foliation between lobule IXa and lobule IXb in the control and its absence in sKO, respectively. **b**, **c** Measurements of areas of total cerebella (**b**) and of posterior lobules VIII, IX and X (**c**) in control and sKO mice at P3, P5 and P30, indicating a severe atrophy in sKO animals. Areas were normalized on mouse body weight and expressed as arbitrary units (AU). P postnatal day, sKO self-knockout. Scale bar: 200 μm. Data are represented as mean ± sem. *p < 0.05, **p < 0.01, ***p < 0.001

We next asked whether the reduction of GCP proliferation observed in Otx2 sKO mice could affect cerebellum development and morphogenesis (Fig. 3). While at P3, no significant defect could be observed, from P5, Otx2 sKO cerebellum appeared smaller, with specific size alteration of the posterior lobules VIII, IX and X, and lacked the fissure of lobule IX characteristic of this stage of development (Fig. 3a, upper panels). At the adult stage, mutant mice exhibited an even more severe phenotype, with their cerebella significantly atrophied (Fig. 3a, lower panels). Quantification showed an overall reduction of cerebellum size of 23% in mutants compared to controls (Fig. 3b). This atrophy was still obvious at P30, suggesting that Otx2 ablation caused an immediate proliferation defect that was maintained over into adulthood. The dramatic reduction in sKO cerebellum size was entirely attributable to an atrophy of posterior lobules VIII, IX and X (Fig. 3c), since no significant change could be observed in the non-Otx2-expressing anterior lobules (data not shown).

Together, these data suggest that Otx2 is necessary for the correct development and morphogenesis of the cerebellum through a cell-autonomous regulation of GCP proliferation in the posterior cerebellum.

### Otx2 controls GCP proliferation independently of Shh

**Fig. 4:**
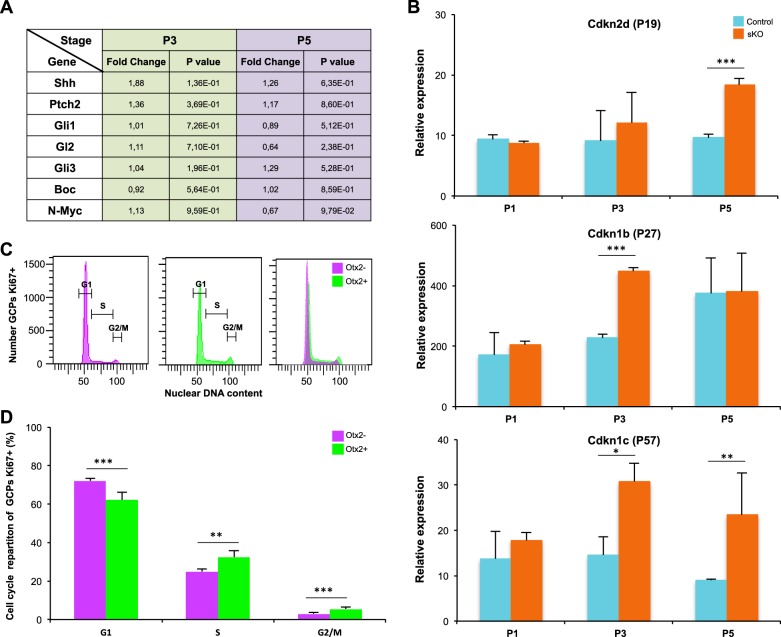
Effect of *Otx2* ablation on Shh signalling pathway and cell cycle gene expression. **a** Regulation of Shh signalling pathway effector genes upon *Otx2* ablation. Expression of the indicated genes was monitored using real-time quantitative PCR and normalized to GAPDH expression. Results are displayed as the fold change between sKO and control cerebella at P3 or P5. None of the tested genes were significantly modified (*p* > 0.05). **b** Relative expression of the cell cycle inhibitors *Cdkn2d (p19), Cdkn1b (p27)* and *Cdkn1c (p57)* in *Otx2* sKO and control cerebella at P1, P3 and P5 indicate upregulation upon *Otx2* ablation. **c** Cell cycle analysis of Otx2+ and Otx2− GCPs. GCPs were purified from *Otx2*^*+/Otx2-GFP*^ cerebella at P7 and analysed by FACS, as in Fig. 1d. Proliferating populations were identified by Ki67 staining and their DNA content was analysed using the DNA-intercalating FxCycle^TM^ fluorescent dye. **d** Distribution of Otx2+ and Otx2− GCPs in each phase of the cell cycle was calculated using the Modfit programme. P postnatal day, sKO self-knockout. Data are displayed as mean ± sem. *p < 0.05, **p < 0.01, ***p < 0.001

To identify the molecular mechanisms underlying Otx2-mediated regulation of GCP proliferation and lobule morphogenesis, we assessed the consequences of *Otx2* sKO on the expression of a series of signalling molecules and cell cycle regulators. Since Shh is required both for the proliferative expansion of GCPs in postnatal EGL and the correct patterning of the cerebellum, we first compared the expression of effectors of the Shh signalling pathway in control and *Otx2*-sKO cerebella at P3 and P5 (Fig. 4). This analysis did not show any significant difference, indicating that Otx2 does not stimulate proliferation through controlling the transcription of Shh pathway effectors (Fig. 4a). In contrast, Cdkn2d (p19), Cdkn1b (p27) and Cdkn1c (p57), three CDK inhibitors (CDKI) involved in blocking the G1 to S-phase transition, were upregulated upon *Otx2* ablation at P3 and/or P5 (Fig. 4b), suggesting that sKO-induced GCP hypoplasia could result from blocking the cell cycle. In accordance with this hypothesis, comparison of the cell cycle distribution of P7 Otx2+ and Otx2− GCPs by FACS analysis showed an increased proportion in G1-phase and a decreased proportion in S-phase in Otx2– cells (Fig. 4c, d).

Together, these data suggest that Otx2 stimulates GCP proliferation independently of Shh by negatively regulating CDKI expression, thus favouring the G1/S transition of the cell cycle.

### Otx2 is critical for long-term maintenance of Shh-dependent medulloblastomas

The present work showing an Shh-independent control of GCP proliferation by Otx2 as well as the lack of Otx2 overexpression in Shh-dependent medulloblastomas^15^ suggests that a high level of Otx2 is not required in tumorigenesis of this subtype. However, whether a normal level of Otx2 is dispensable or not, in this process, has never been investigated. To address this, we created a novel in vivo model of Shh-dependent medulloblastomas by directing constitutive expression of an activated form of Smoothened (SmoM2)^24^ in Otx2+ GCPs (*Otx2*^*CreERT2/+*^*;R26*^*SmoM2/SmoM2*^ line) and next investigated Otx2 function in tumour cell proliferation by simultaneous Otx2 ablation (*Otx2*^*CreERT2/flox*^*;R26*^*SmoM2/SmoM2*^ sKO line).

**Fig. 5:**
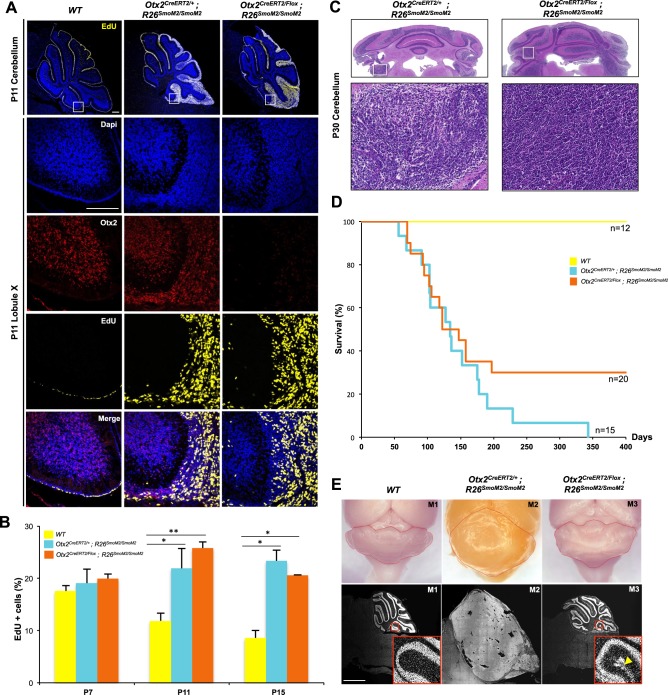
Effect of *Otx2* ablation on Shh medulloblastoma formation and their maintenance. *Otx2*^*+/+*^ (WT), *Otx2*^*CreERT2/+*^*;R26*^*SmoM2/SmoM2*^ (allowing *SmoM2* conditional expression) and *Otx2*^*CreERT2/flox*^*;R26*^*SmoM2/SmoM2*^ (allowing *SmoM2* conditional expression and concomitant *Otx2* ablation) mice were injected with tamoxifen at P2 and P5 and analysed at different stages. **a** Detection of proliferating cells in P11 cerebella. Mice were injected with EdU 1 h before euthanasia. Detection of Otx2 expression (red), EdU incorporation (yellow) and nuclei (blue) on lobule X (white boxes) shows massive hyperplasia, regardless of the presence of Otx2. **b** FACS quantification of EdU+ cells at P7, P11 and P15 in the GCP fraction of the three colour-coded genotypes. **c** Upper panels: Hematoxylin and eosin staining of posterior cerebella at P30 (coronal sections) showing the presence of classical medulloblastoma. Lower panels: Magnification of boxed areas in the above images. **d** Survival curves of *Otx2*^*CreERT2/+*^*;R26*^*SmoM2/SmoM2*^ and *Otx2*^*CreERT2/flox*^*;R26*^*SmoM2/SmoM2*^ mice showing divergence after 6 months. **e** Long-term follow-up of *WT*, *Otx2*^*CreERT2/flox*^*;R26*^*SmoM2/SmoM2*^ and *Otx2*^*CreERT2/flox*^*;R26*^*SmoM2/SmoM2*^ mice sampled after this divergence. M1: *WT*, 609 days; M2: *Otx2*^*CreERT2/+*^*;R26*^*SmoM2/SmoM2*^, 343 days; M3: *Otx2*^*CreERT2/flox*^*;R26*^*SmoM2/SmoM2*^; 605 days. Upper panels: Bright-field dorsal views of posterior brain (red lines delimit the cerebellum). Lower panels: Hoechst staining of mid-sagittal cerebellar sections showing dramatic tumour regression in *Otx2*^*CreERT2/flox*^*;R26*^*SmoM2/SmoM2*^ mice. Insets: Magnification of boxed areas at the level of lobules IX and X. Yellow arrowhead points to EGL-like persisting tumour remnants in external position in *Otx2*^*CreERT2/flox*^*;R26*^*SmoM2/SmoM2*^ cerebellum. P postnatal day. Scale bar: 300 μm (**a**) and 1000 μm (**e**). Data are displayed as mean ± sem. *p < 0.05, **p < 0.01

Tamoxifen induction of SmoM2 in the early postnatal cerebellum of *Otx2*^*CreERT2/+*^*;R26*^*SmoM2/SmoM2*^ animals resulted in rapid overproliferation of GCPs followed by the formation of neoplastic lesions restricted to the posterior cerebellum, reflecting Otx2 expression pattern (Fig. 5). While at P11, the majority of cerebellar GCPs had already stopped dividing in the controls, their EGL being reduced to a few cell rows, *Otx2*^*CreERT2/+*^*;R26*^*SmoM2/SmoM2*^ mice displayed EGL hyperplasia and extended GCP proliferation, as shown by the persistence of several rows of EdU+ cells in posterior lobules (Fig. 5a). Quantification of Ki67+ cells by FACS analysis indicated that more than 75% of GCPs were still proliferating at P16 (data not shown), of which 20% were EdU+ (Fig. 5b). By P30, these mice displayed diffuse tumours encompassing the posterior cerebellum. Tumour proliferation was often observed in the lepto-meningeal space, without invasion of the cerebellar parenchyma. Histological analysis revealed the presence of ‘small round blue’ pleomorphic cells with hyperchromatic nuclei, often associated with the ‘classical’ forms of medulloblastoma in humans, but no cell wrapping or marked nucleoli, as seen in anaplastic or large-cell medulloblastomas, respectively (Fig. 5c). As expected, these bona-fide type 2 medulloblastomas did not display Otx2 overexpression (Sup. Figure 1).

Simultaneous ablation of *Otx2* in the *Otx2*^*CreERT2/flox*^*;R26*^*SmoM2/SmoM2*^ sKO line neither affected early GCP expansion and EGL hyperplasia at P11 and P15 (Fig. 5a, b) nor the extent of tumoral development at P30 (Fig. 5c), or tumour size before this stage (sup. Fig. 2A). In this context, no significant difference in CDKI expression could be observed (sup. Fig. 2B), suggesting that SmoM2-induced hyperproliferation could not be counteracted by *Otx2* ablation. In both lines, medulloblastomas developed in 100% of mice, with a median survival of 130.5 days in *Otx2*^*CreERT2/+*^*;R26*^*SmoM2/SmoM2*^ and 122 days in *Otx2*^*CreERT2/flox*^*;R26*^*SmoM2/SmoM2*^ mice (Fig. 5d). At 5 months, survival rates were similar in the two lines (40% and 45%, respectively). Together, these results indicate that *Otx2* is not required to initiate medulloblastoma formation in Otx2+ granule cell precursors under constitutive SmoM2 stimulation.

In contrast, long-term follow-up of tumour development revealed striking effects of *Otx2* ablation. While tumours kept growing and invaded the whole cerebellum space in *Otx2*^*CreERT2/+*^*;R26*^*SmoM2/SmoM2*^ mice, leading to a survival rate of 0% at 12 months, 30% of *Otx2*^*CreERT2/flox*^*;R26*^*SmoM2/SmoM2*^ KO mice survived normally (Fig. 5d, e). Furthermore, tumours dramatically regressed in these mice leaving only medulloblastoma remnants in external position at 18 months (Fig. 5e), indicating that *Otx2* ablation was sufficient to counteract SmoM2 mitogenic activity and long-term tumour maintenance.

Together, these data indicate that, while Otx2 does not act as a driver of type 2 medulloblastoma tumoral transformation, as suggested before, it unexpectedly plays a mandatory function in sustaining these tumours over time.

## Discussion

Here we have addressed the physiological function of Otx2 in the context of the developing organism. We report that *Otx2* is expressed in a subpopulation of GCPs with higher proliferative index, located in the most-posterior part of the postnatal cerebellum and that *Otx2* ablation in these cells impairs their proliferation through a Shh-independent, direct effect on the cell cycle. We have next assessed whether Otx2 is truly devoid of role in the context of type 2 medulloblastoma development by directing constitutive expression of an activated form of smoothened in Otx2+ GCPs of the postnatal cerebellum. We demonstrate that although *Otx2* ablation in this context does not prevent the formation of tumours, it strongly impairs their long-term maintenance, demonstrating for the first time, the critical role of this factor in regulating proliferation and tumorigenesis in the perinatal cerebellum. Our data point to the existence of two independent proliferation pathways in GCPs: a Shh-dependent pathway that is dominant for GCP proliferation and type 2 MB tumour formation, and an Otx2-dependent pathway that is critical for proliferation of Otx2-expressing GCP subpopulation, and that becomes mandatory for long-term maintenance of type 2 MB tumour growth.

### Otx2 exerts a direct, Shh-independent pro-proliferative role in a subset of granule cell precursors

Here we have disclosed a novel important function of Otx2 in regulating proliferation in a specific population of GCPs in vivo. *Otx2*-KO GCPs display an increased expression of CDK inhibitors that block the G1/S transition of the cell cycle, suggesting that Otx2 stimulates GCP proliferation by repressing S-phase entry brakes. This role of Otx2 in GCP proliferation poses the question of its relationship with Shh signalling, the major pathway responsible for GCP transit amplification. In postnatal GCPs, Shh stimulates cell proliferation through upregulation of the transcription factor N-Myc, itself stimulating cyclin-D2 expression^8,25^. By contrast, *Otx2* loss of function has no effect on N-Myc and cyclin-D expression, suggesting that Shh and Otx2 control GCP proliferation through parallel and complementary pathways. Our finding that *Otx2* ablation does not affect the expression of Shh downstream effectors, including its main target *Gli1*, confirms that Otx2 is not part of the Shh signalling network. Of note, Shh^−/−^ mice are not totally devoid of cerebellum, and anti-Shh-treated mice still possess an EGL, supporting the idea that GCP proliferation relies on additional mechanisms^26,27^. We suggest that Otx2 acts through the repression of CDK inhibitors, and particularly p27, which is the CDK inhibitor expressed at the highest and most significant level in postnatal GCPs^28^. The action of p27 appears to be dominant over proliferative signalling as even in the presence of saturating amounts of Shh, granule cells that progressively accumulate sufficient levels of p27 stop dividing and differentiate. Negative control of such a strong cell cycle inhibitor by Otx2 therefore appears as a powerful way to increase the proliferation rate and extend the proliferation window of GCPs. This might explain why, despite entering the EGL later, posterior GCPs generate as many granule cell progeny as anterior GCPs, in a shorter period, providing the cerebellum its homogeneous histology all along the anterior–posterior axis. Whether the regulation of p27 transcription by Otx2 is direct or not remains to be investigated.

### *Otx2* expression is dispensable in vivo for early, but not long-term Shh-induced tumour growth

The existence of a novel, Shh-independent control of GCP proliferation by Otx2 we report here suggests, as previously assumed, that Otx2 is not involved in tumorigenesis of type 2 medulloblastomas. To address this hypothesis, we have developed a novel tumour model of type 2 medulloblastoma by inducing constitutive Shh signalling in Otx2+ GCPs of the postnatal cerebellum and assessing the effect of *Otx2* loss of function in this model. It was previously shown that permanent activation of the Shh pathway requires acquisition of a granular cell fate to promote tumour formation^29^. Consistently, in our model, we observe a massive proliferation of the external layer of the developing posterior cerebellum while the neighbouring choroid plexus, which also expresses Otx2, hence SmoM2, remains perfectly normal. Of note, the localization, growth rate and histology of tumours induced in Otx2+ GCPs correspond to bona-fide classical type of Shh medulloblastoma. The fact that 100% of the mice develop pre-neoplastic lesions, regardless of the presence of Otx2, indicates that Otx2 is dispensable for tumoral initiation and suggests that, at early stages of tumour development, the amount of Shh signal delivered by SmoM2 constitutive expression overrides the Otx2 parallel pathway.

In contrast, prolonged absence of Otx2 causes a totally different evolution, as specific regression of tumour development is observed in sKO animals, therefore unravelling an unexpected, mandatory function for Otx2 in sustaining long-term maintenance of type 2 medulloblastoma in vivo. These findings suggest that Shh signalling no longer fully operates in these aging tumours, which may then progressively become dependent on the Otx2 parallel pathway to sustain proliferation. During normal development, EGL cells naturally lose their responsiveness to Shh when they differentiate. This has been attributed to the presence, in the postnatal EGL, of extracellular matrix molecules and growth factors able to interfere with Shh-induced proliferative response^9^. It is conceivable that these interfering cascades also operate in SmoM2-induced aging tumours, albeit with a delay due to the overactivation of Shh signalling. A minimal level of Otx2 could then become necessary to maintain tumour cell proliferation and block the mechanisms that normally allow GCPs to stop proliferating. Again, inhibition of p27 accumulation in aging tumour cells appears as a plausible mechanism to prevent their exit from the cell cycle.

### Why is human Shh medulloblastoma the only type that does not show Otx2 overexpression?

We show here that Otx2 is an important regulator of GCP proliferation. It seems contradictory, at first, that Otx2 overexpression is found in all types of medulloblastomas except in Shh type, as the latter is known to originate from GCPs. Again, the existence of two parallel and independent pathways controlling GCP proliferation, as we report here, brings a simple explanation to this apparent paradox. In such a situation, deregulation of one of these two pathways would be sufficient to promote tumour formation. In accordance with this proposal, overactivation of the Shh pathway in the present *Otx2*^*CreERT2/flox*^*;R26*^*SmoM2/SmoM2*^ murine model is sufficient to induce type 2 medulloblastomas in the complete absence of Otx2. Extrapolation of this observation to human type 2 medulloblastomas would imply that deregulation of SHH signalling does not require more than normal OTX2 levels to ensure tumour cell proliferation. Conversely, one would expect that in other types of medulloblastoma with granule precursor as the cell of origin, but where the Shh pathway is not deregulated, proliferation would mostly rely on the Otx2 alternative pathway. This might explain why amplification and/or overexpression of *Otx2* need to be selected, together with other genetic alterations, to ensure sustained proliferation of Shh-independent medulloblastomas. In accordance with this model, *OTX2* overexpression is observed in more than 75% of medulloblastomas, and is among the most frequent targets of focal copy number gain reported in group 3 and group 4 medulloblastomas^15^.

**Fig. 6:**
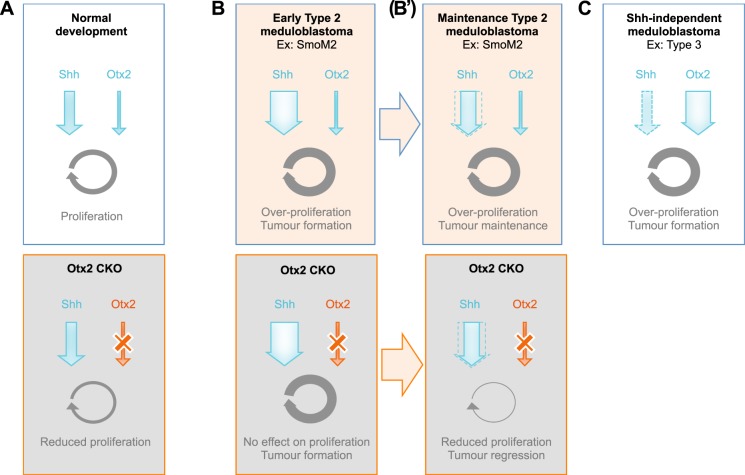
Model for Shh and Otx2-mediated regulation of cerebellar GCP proliferation in the context of normal development and medulloblastoma formation. **a** During normal development, both Shh and Otx2 stimulate GCP proliferation through parallel signalling pathways. Ablation of *Otx2* in this context, by shutting down of one of these pro-proliferative pathways, significantly impairs GCP proliferation, and leads to posterior cerebellum atrophy. **b** In the context of SmoM2 constitutive activation, forced Shh stimulation results in GCP overproliferation and the formation of type 2 medulloblastomas. This occurs whether Otx2 is present or absent, indicating that overactivation of the Shh pathway is dominant on the Otx2 pathway and is sufficient to promote tumour initiation in this context. **b’** As they age, tumour cells become less responsive to Shh pathway signalling and more dependent on Otx2 signalling to sustain proliferation. Ablation of *Otx2* in this context impairs cell proliferation and causes tumour regression. **c** In other (Shh-independent) types of medulloblastoma originating from GCPs, where the Shh pathway is not overactivated, such as type 3 medulloblastoma, proliferation mainly relies on the Otx2 alternative pathway: overexpression of Otx2 is frequently selected to ensure sustained proliferation of tumour cells

A model recapitulating our findings and how Shh and Otx2 pathways control normal and tumoral proliferation of GCPs is presented in Fig. 6.

### Otx2 as a target to inhibit medulloblastoma progression

The development of small-molecule antagonists of the SHH pathway has provided new perspectives for the treatment of Shh-dependent medulloblastomas^30,31^. In particular, SMO inhibitors have been shown to effectively suppress this subgroup of tumours in clinical trials. However, drug resistance or long-term disease revival frequently occur, urging the need for novel therapeutic agents that may act on downstream elements of the SHH pathway, or on other tumorigenic pathways. In this regard, it is conceivable that drugs aiming at reducing *OTX2* expression could be useful for long-term treatment of Shh-dependent type 2 medulloblatomas, since, as we show here, OTX2 acts in parallel of the SHH pathway and becomes mandatory for long-term maintenance of SmoM2-induced tumours in mice. Unfortunately, specific OTX2 inhibitors are not presently available, nor are understood the signals that positively regulate its expression in GCPs. It will be of great benefit to study the regulatory pathways that control *OTX2* expression in order to develop specific drugs to reduce *OTX2* gene activity in tumours. So far, only in vitro knockdown of Otx2 using siRNA and retinoic acid treatment have been achieved^32^, but demonstration of their feasibility and efficiency in vivo is still awaited. The data presented here urge to tackle these critical questions, as OTX2 appears as an attractive candidate for molecularly targeted therapy in a large fraction of medulloblastomas.

## Materials and methods

### Mouse breeding and injections

All animal experimentations were approved by the local and French Ministry ethical committees. All mouse strains were maintained in 129/Sv background. *Otx2*^*+/Otx2-GFP*^*, Otx2*^*CreERT2/+*^ (control) and *Otx2*^*CreERT2/flox*^ (sKO) mouse lines were described previously^12^. *Gt(ROSA)26Sor*^*tm1(Smo/EYFP)Amc/J*^ (R26^SmoM2/SmoM2^) mice were obtained from the Jackson Laboratory and crossed with *Otx2*^*CreERT2/+*^ or *Otx2*^*CreERT2/flox*^ to get Otx2^CreERT2/+^;R26^SmoM2/SmoM2^ and Otx2^CreERT2/flox^;R26^SmoM2/SmoM2^, respectively. Mice of either sex were used. Genotypes were determined by PCR analysis using primers described in Supplementary Table 1. Intraperitoneal injection of tamoxifen (40 µg/g of body weight, Sigma-Aldrich, St Louis, MO, USA) was performed at P1 or at P2 and P5 unless otherwise indicated. Intraperitoneal injection of 5-ethynyl-2′-deoxyuridine (EdU) (25 µg/g, Sigma-Aldrich) was performed 1 h before euthanasia. Tamoxifen injections were performed blindly on litters containing animals of unknown genotypes. At least three biological samples were used per genotype and per stage to ensure reproducibility.

### Granular cell precursor purification and fluorescence-activated cell sorting (FACS) analysis

Cerebella were dissociated using papain/DNAse enzymatic digestion following the manufacturer’s instruction (Worthington, Lakewood, NJ, USA) and GCPs were purified using Percoll gradient separation as previously described^33^. For FACS analysis, GCPs were fixed for 40 min at 4 °C in TF fixation buffer (transcription factor buffer set, BD Biosciences, Franklin Lakes, NJ, USA), incubated for 40 min at 4 °C with anti-GFP (Alexa Fluor (AF) 488-conjugated; 1:500; Invitrogen, Waltham, MA, USA) and anti-Ki67 (PE-conjugated, 1:600; Biolegend, San Diego, CA, USA) antibodies. EdU was revealed using Click-it EdU AF647 Flow Cytometry Assay Kit (Invitrogen). Cell cycle repartition was analysed using FxCycle^TM^ DNA staining (Invitrogen) and the percentage of cell in each phase was calculated using the Modfit mathematical modelling software (version 3.3, VSH, Topsham, ME, USA). Cells were analysed using a LSR Fortessa flow cytometer (BD biosciences).

### RNA isolation and qRT-PCR

Cerebella were triturated in TRI-reagent (Sigma-Aldrich). Total RNA was extracted using miRNeasy Mini Kit (Qiagen, Hilden, Germany). First-strand cDNA was synthesized using Super Script III reverse transcriptase (Invitrogen). For real-time PCR, 25 ng of cDNA was used per reaction and amplified using PowerSYBR Green PCR mix and Step One Plus apparatus and software (Applied Biosystems, Foster City, CA, USA). Gene to GAPDH ratios were determined using ΔΔCT = 1+E_GAPDH_^(CTsKO − CTcontrol)^/1+E_gene_^(CTsKO − CTcontrol)^ formula, where E represents the primer efficiency. Primers are listed in Sup. Table 1.

### Immunocytochemistry and histological analysis

For immunocytochemistry, cerebella were fixed in 4% paraformaldehyde overnight at 4 °C, protected in 30% PBS-sucrose and frozen in Tissue-Tek OCT at −80 °C (Fisher Scientific, Waltham, MA, USA). Sagittal sections of 12 µm (±300 µm from the midline) were cut on a Microm HM550 cryostat, mounted on SuperFrost+slides (Fisher Scientific) and blocked 1 h in PBST (PBS with 0.4% gelatin, 2% DMSO, 0.2% Triton X-100 and 10% donkey serum). Sections were incubated overnight at 4 °C with primary antibodies and 1 h at RT with secondary antibodies. Primary antibodies were goat anti-Otx2 (1:200, R&D Systems, Minneapolis, MN, USA), rabbit anti-Ki67 (1:100, Thermo Scientific, clone SP6), rabbit anti-GFP (1:500, Abcam, Cambridge, UK). Secondary antibodies were anti-goat Cy3 (1:1000) and anti-rabbit Alexa Fluor 488 (1:1000). EdU was revealed using Click-it Edu Alexa Fluor 647 Imaging kit (Invitrogen). Nuclei were counterstained with Hoeschst 33342 (40 µg/ml, Promega, Madison, WI, USA). The percentage of EdU+ cells was estimated by counting the total number of nuclei and of EdU+ cells in three different regions of the EGL of lobules I, II, III, VIII, IX and X. For pathological examination, tissue blocks were paraffin-embedded, cut into coronal 4-μm sections and stained with hematoxylin and eosin (H&E) using standard methods. Images were generated on Zeiss 710 Confocal (Zeiss, Oberkochen, Germany) and Leica M205 FA microscopes. Areas were estimated using ImageJ software (NIH, Bethesda, MA, USA) and normalized to mouse body weight.

### Statistical analysis

At least three biological samples per genotype and per stage were used to ensure reproducibility. Statistics were performed using two-tailed Student’s *t* test with a *p* value <0.05 considered as significant. Results are presented as the mean ± sem. Variation between compared groups was found to be similar using F-testing. Mice survival was analysed using Kaplan–Meier curves and the significance of the results was assessed by log-rank test.

## Electronic supplementary material

Supplemental Figure 1

Supplemental Figure 2

Supplemental Table 1
