# Supplementary material for: Otx2 promotes granule cell precursor proliferation and Shh-dependent medulloblastoma maintenance in vivo
Source: Oncogenesis. 2018 Aug 13;7(8):60. doi: 10.1038/s41389-018-0070-6 (PMC6087714; doi:10.1038/s41389-018-0070-6)
Supplement: Supplementary file 1 — Supplemental Figure 1 [file 41389_2018_70_MOESM1_ESM.pdf]

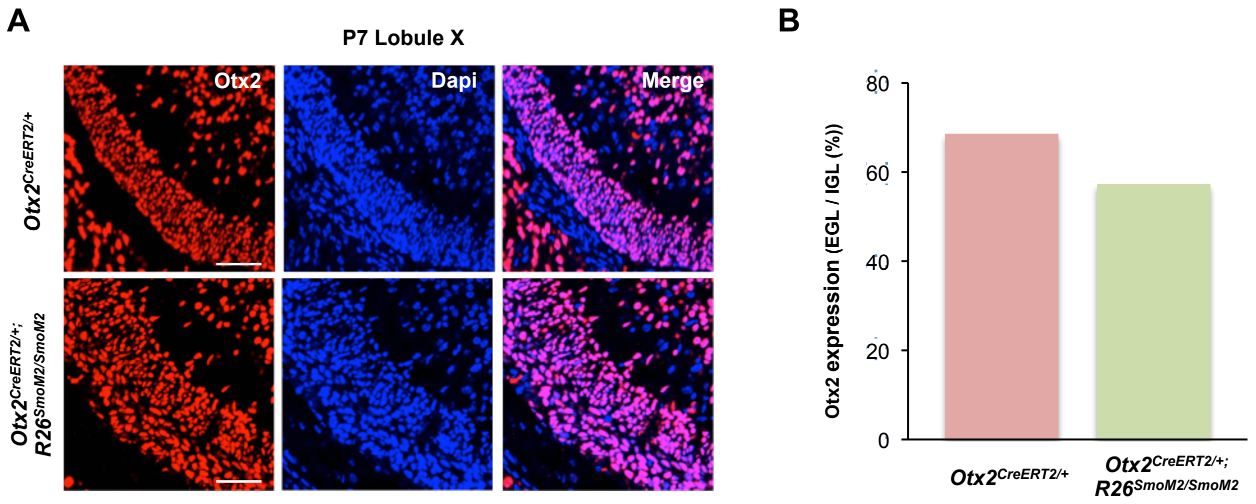

Supplementary Figure 1: Quantification of Otx2 protein expression in *Otx2*$^{CreERT2/+}$ versus *Otx2*$^{CreERT2/+;}$ *R26*$^{SmoM2/SmoM2}$ cerebellum. (A) Mice were injected with tamoxifen at P1 and Otx2 expression was assessed by immunofuorescence detection (red) on sagittal sections of lobule X at P7. DAPI staining was used to detect nuclei (blue). (B) Relative quantification of Otx2 in *Otx2*$^{CreERT2/+}$ versus *Otx2*$^{CreERT2/+;}$ *R26*$^{SmoM2/SmoM2}$ EGL. Otx2 and Dapi expression were quantified on defined areas of EGL and IGL. In each area, Otx2 expression was first normalised to Dapi expression to compare staining intensity levels between different slides. Relative expression of Otx2 in EGL was then measured for each genotype as the ratio of Otx2 expression in EGL /Otx2 expression in IGL (that is not affected by tumour development). Scale bar: 40 µm.
