# Supplementary material for: Otx2 promotes granule cell precursor proliferation and Shh-dependent medulloblastoma maintenance in vivo
Source: Oncogenesis. 2018 Aug 13;7(8):60. doi: 10.1038/s41389-018-0070-6 (PMC6087714; doi:10.1038/s41389-018-0070-6)
Supplement: Supplementary file 2 — Supplemental Figure 2 [file 41389_2018_70_MOESM2_ESM.pdf]

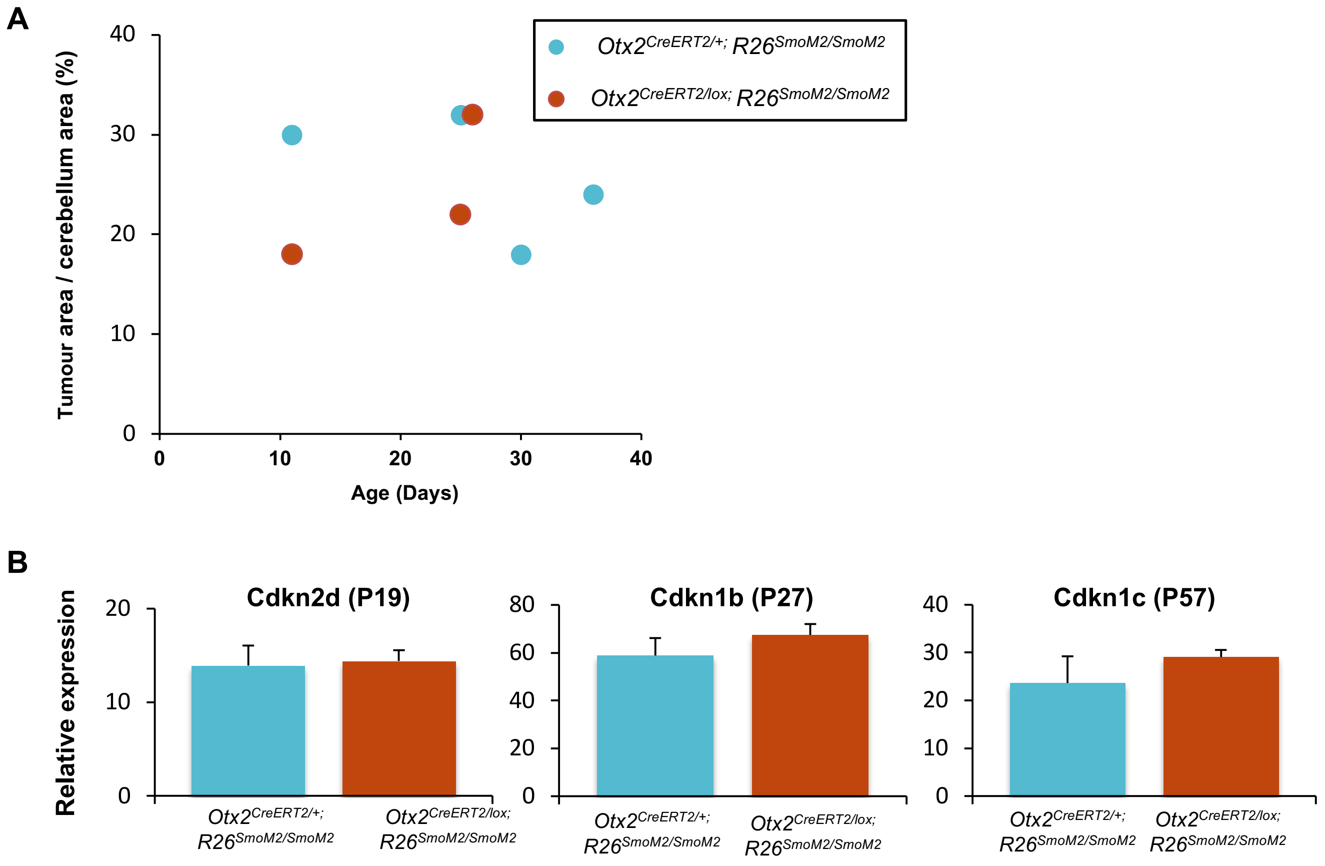

Supplementary Figure 2: Assessment of tumor size and expression of cell cycle inhibitors in *Otx2$^{CreERT2/+}$; R26$^{SmoM2/SmoM2}$* and *Otx2$^{CreERT2/lox}$; R26$^{SmoM2/SmoM2}$* (Otx2cKO) mice. (A) Quantification of tumour size in the cerebellum of *Otx2$^{CreERT2/+}$; R26$^{SmoM2/SmoM2}$* versus *Otx2$^{CreERT2/lox}$; R26$^{SmoM2/SmoM2}$* mice in the first postnatal month after SmoM2 induction. Mice were injected with tamoxifen at P1 and P5 and tumour development was assessed at different times by measuring tumour area and normalizing it to total cerebellum area on cerebellum sagital sections . Two-tailed Student's t-test was performed and indicate that tumour sizes are not significantly different in the two lines (p value = 0.67). (B) QPCR analysis of the expression of the cell cycle inhibitors *Cdkn2d (p19), Cdkn1b (p27) and Cdkn1c (p57)* in *Otx2$^{CreERT2/+}$; R26$^{SmoM2/SmoM2}$* and *Otx2$^{CreERT2/lox}$; R26$^{SmoM2/SmoM2}$* cerebella at P5 indicates no significant difference between the two genotypes (p values > 0.05).
