# Supplementary material for: Otx2 promotes granule cell precursor proliferation and Shh-dependent medulloblastoma maintenance in vivo
Source: Oncogenesis. 2018 Aug 13;7(8):60. doi: 10.1038/s41389-018-0070-6 (PMC6087714; doi:10.1038/s41389-018-0070-6)
Supplement: Supplementary file 3 — Supplemental Table 1 [file 41389_2018_70_MOESM3_ESM.pdf]

Supplementary table 1: List of primer sequences used for mice genotyping and for RT-qPCR analysis.

Genes	Primers	Sequences
**Genotyping Primers**		
Otx2-CreERT2	Foward	CCAAATCTACCCACCAAGGA
	Reverse	GACCGACGATGAAGCATGTT
Otx2-Flox	Foward	GAACAAACGTCCCTGTGGTG
	Reverse	AGGAGCCACAATTCCCATC
Otx2-GFP	Foward	GCTGGCTCAACTTCCTACT
	Reverse	ACCCTCTCCACTGACAGAA
Rosa26-Stop flox	Foward	AAAGTCGCTCTGAGTTGTTAT
	Reverse	GCGAAGAGTTTGTCCTCAACC
**Real time qPCR Primers**		
Otx2	Foward	CCAAATCTACCCACCAAGGA
	Reverse	AGAGCTTCCAGAACGTCGAG
Cdkn2d (p19)	Foward	AATGTGACCCAAGGCCACT
	Reverse	TTTCCTCTTTTGTTGACAAGTAACC
Cdkn1b (p27)	Foward	GAGCAGTGTCCAGGGATGAG
	Reverse	TCTGTTCTGTTGGCCCTTTT
Cdkn1c (p57)	Foward	CGCAAACGTCTGAGATGAGT
	Reverse	CCCAGAGTTCTTCCATCGTC
Shh	Foward	CCAACGTAGCCGAGAAGACC
	Reverse	CTTGTCTTTGCACCTCTGAGTC
Patch2	Foward	CCTAGAACAGCTCTGGGTAGAAGT
	Reverse	CCCAGCTTCTCCTTGGTGTA
Gli1	Foward	CAGGGTCCCAGGGTTATGG
	Reverse	AGGTCGAGGCTGGCATCAG
Gli2	Foward	TCCCCGACCCAGGGAAAAA
	Reverse	CCCTCCTGGTGTCTCATGTC
Gli3	Foward	AGAGTGCCTCCAGGTGAAGA
	Reverse	GTTGCTGATGGGGGACTGTT
Boc	Foward	GCTACCAGTGCCATACCTCC
	Reverse	CGGGCCTCTCATATACACGG
Mycn (Nmyc)	Foward	TGTGTCTGTTCCAGCTACTGC
	Reverse	CTTCCTCCTCGTCATCCTCA
GAPDH	Foward	CATGGCCTTCCGTGTTCCTA
	Reverse	TGCCTGCTTCACCACCTTCT
